# Sperm Cell Membranes of Bulls and Bucks Associated with Sperm Fertility and Freezability

**DOI:** 10.3390/ani15223248

**Published:** 2025-11-09

**Authors:** Seher Simsek, Mustafa Hitit, Mustafa Bodu, Erdogan Memili

**Affiliations:** 1 Ministry of Agriculture and Forestry, Republic of Türkiye, General Directorate of Livestock, Ankara 06060, Türkiye; seher.yirtici@tarimorman.gov.tr; 2 Cooperative Agricultural Research Center, College of Agriculture, Food, and Natural Resources, Prairie View A&M University, Prairie View, TX 77446, USA; 3 Department of Reproduction and Artificial Insemination, Faculty of Veterinary Medicine, Selçuk University, Konya 42075, Türkiye

**Keywords:** sperm, fertility, cryopreservation, membrane, freezability, bull, buck

## Abstract

**Simple Summary:**

Changes caused by cryopreservation can damage membrane integrity and reduce sperm viability. A better understanding of sperm membrane characteristics, including factors like the cholesterol–phospholipid ratio and membrane phase transition temperature, can help identify biomarkers for fertility and freezability. This knowledge also supports the design of improved semen extenders, which protect sperm during storage and enhance assisted reproductive technologies (ART) in animals and humans. Studying sperm membranes at the molecular and cellular levels is therefore key for advancing fertility research, improving breeding programs, and supporting the conservation of endangered species.

**Abstract:**

Consisting of phospholipids, sperm membranes surround the head and tail, playing essential roles in maintaining cellular structural integrity and functions. Their characteristics directly influence sperm fertility and cryopreservation outcomes. This minireview provides a summary of how sperm fertility and freezability are affected by the characteristics of its cell membranes. The primary emphasis is on the molecular and cellular anatomy as well as the physiology of sperm membranes and their attributes associated with fertility determinants or biomarkers for fertility and freezability. It also explores how this knowledge can guide the development of extenders to improve sperm freezability and enhance reproductive technologies in mammals. By providing integrity, fluidity, and selective permeability, the membranes play vitally important roles in sperm motility, which is required for successful fertilization. Cryopreservation, which involves freezing and thawing of sperm for storage or ART, alters the integrity and functionality of the sperm membranes. Sperm freezability, its viability following freezing and thawing, is influenced by several properties of the sperm cell membranes, such as lipid composition, cholesterol content, and structures and functions of the membrane proteins. This review provides concise information about the nature of sperm membranes. It highlights the importance of understanding specific biophysical and biochemical features, including lipid composition, protein distribution, and membrane phase behavior. Particular attention is given to parameters such as the cholesterol–phospholipid ratio and membrane phase transition temperature (Tm). A deeper understanding of these factors can contribute to the identification of reliable fertility biomarkers and the optimization of cryopreservation techniques used in ART and animal breeding programs. Furthermore, this review underscores the need for comprehensive investigations into the molecular and cellular architecture of sperm cells. Such studies are essential for advancing both fundamental and applied aspects of reproductive biology in food-producing animals, endangered species, and humans.

## 1. Introduction

Livestock farming plays a vital role in the agricultural sector by contributing to food security and economic security [1,2]. Efficient reproduction is essential for sustainable and profitable production, relying on fertility in both males and females [1,3]. Male fertility is the ability of a male to produce sufficient numbers of viable and motile sperm, which is crucial for genetic dissemination and productivity [4,5]. The fertility of bulls, bucks, and rams is influenced by genetics, hormone levels, reproductive anatomy, nutrition and environmental factors [6,7]. Semen production and sperm quality are key traits for breeding success and genetic improvement in livestock in bulls, rams, and goats [5,8].

Molecular and cellular anatomy and the physiology of mammalian sperm, along with species-specific similarities and differences, particularly in bulls and bucks, provide key insights into reproductive strategy, fertility, and reproductive health [4,9,10]. This knowledge supports the development of reproductive management techniques and interventions for cattle and goats. Such advances can improve genetic selection, breeding programs, and overall animal productivity [8,11,12]. However, a clearer understanding of species-specific sperm membrane characteristics and their relationship to fertility and freezability remains a critical gap that this review aims to address. The term freezability is used to describe the capacity of sperm cells to tolerate freezing and thawing procedures without significant loss of viability, motility, or fertilizing ability, which are critical indicators of post-thaw sperm quality.

**Figure 1:**
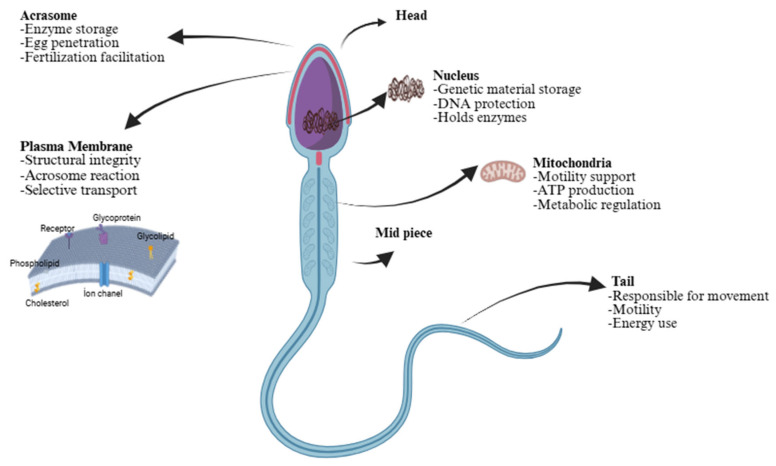
The anatomy and physiology of mammalian sperm membranes; the figure illustrates the structural organization and functional components of a mammalian sperm cell. The head region contains the acrosome, which stores enzymes facilitating egg penetration and fertilization, and the nucleus, which protects and stores genetic material. The plasma membrane maintains structural integrity, supports selective transport, and mediates the acrosome reaction. The midpiece contains numerous mitochondria responsible for ATP production, metabolic regulation, and motility support. The tail enables movement and propels the sperm toward the egg through coordinated flagellar motion.

Spermatozoa have a distinct structure comprising a head, mid-piece, and tail [13,14]. The spermatozoon consists of a head containing the nucleus and the acrosome, which contains enzymes required for fertilization, including hyaluronidase, acrosin, zona pellucida-binding proteins, phospholipases, neuraminidase, and trypsin-like proteases. The tail (flagellum) propels the sperm forward [15] (Figure 1). Sperm fertility has been directly influenced by acrosome integrity and enzymes. For instance, the midpiece supplies energy because it is rich in mitochondria [16]. Thus, the structural integrity and functional specialization of sperm anatomy are fundamental determinants of male fertility.

For fertility, energy is required, which sperm obtain from their mitochondria to reach and fertilize the oocyte. It is influenced by a number of factors, including the integrity of the flagellum, ionic balance, membrane fluidity, and energy availability. Among these, energy supply, particularly in the form of ATP, is a key determinant of this process [17]. Mitochondria in the midpiece generate ATP through oxidative phosphorylation, providing the energy essential for sperm motility and fertilization. Metabolites such as pyruvate and lactate enter the mitochondrial matrix, where they are oxidized through the tricarboxylic acid (TCA) cycle, producing reduced cofactors, NADH and FADH_2_. These cofactors donate electrons to the mitochondrial electron transport chain, creating a proton gradient across the inner mitochondrial membrane. The return flow of protons through ATP synthase drives the phosphorylation of ADP to ATP. Oxygen serves as the final electron acceptor, forming water. The ATP produced is then utilized to power the flagellar movement of the sperm, enabling progressive motility and successful navigation through the female reproductive tract [4,18,19].

Fertilization is a complex and highly coordinated process that begins with the fusion of a motile sperm and a mature oocyte, requiring successful sperm navigation, binding to the zona pellucida, and the acrosome reaction [20]. Sperm motility is driven by dynein ATPase activity, which powers the sliding of microtubules within the flagellum to produce the wave-like movements essential for progression through the female reproductive tract [21]. This motility and fertilization capacity are acquired during sperm maturation in the epididymis [22]. Mammals share fundamental sperm characteristics at the molecular and cellular levels, with variations in sperm size, shape, and seminal fluid composition. Understanding these structural and molecular mechanisms supports the main goal of this review, which is to highlight how the biophysical and biochemical properties of sperm membranes play a crucial role in identifying fertility biomarkers and enhancing ART [23,24]. Recent reviews have focused on either lipid compositional changes during cold acclimation or cryoprotectant chemistry and application. In contrast, this review centers on the biophysical and biochemical features of sperm membranes, emphasizing parameters such as lipid composition, cholesterol–phospholipid ratio, and membrane phase Tm. By linking these factors to sperm function, fertility, and freezability, this review provides a mechanistic and application-oriented perspective that advances our current understanding of reproductive biology and cryopreservation.

## 2. Sperm Cell Membranes and Fertility in Mammals

### 2.1. Lipid Composition and Membrane Dynamics

**Figure 2:**
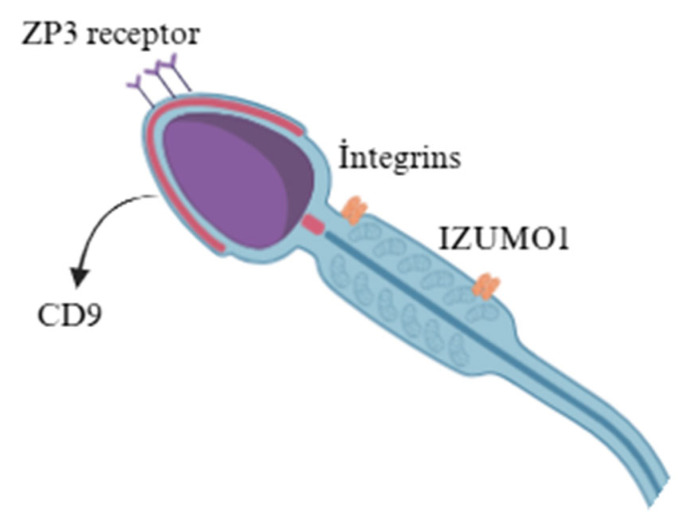
Fertilization-related proteins of mammalian sperm; the figure depicts key membrane proteins involved in sperm–egg recognition and fusion. The ZP3 receptor mediates the initial binding of sperm to the zona pellucida of the oocyte, while CD9, located on the oocyte membrane, facilitates membrane fusion during fertilization. Integrins contribute to sperm–egg adhesion and signal transduction, and IZUMO1, a sperm membrane protein, is essential for gamete fusion and interaction with its oocyte counterpart, JUNO.

The sperm plasma membrane comprises various lipids, including phospholipids, glycolipids, and sterols. Phospholipids provide the basic structural framework of the membrane by forming a bilayer in which their hydrophobic fatty acid tails orient inward and their hydrophilic heads face outward, creating a semi-permeable, dynamic barrier [25,26]. In addition, during testicular and epididymal maturation, the composition of the sperm plasma membrane evolves, with spermatozoa gaining the remarkable capacity to fertilize the egg. The head sperm has a higher concentration of specific proteins, particularly IZUMO1, ZP3 receptors, and integrins and lipids that are involved in egg binding and acrosomal reactions [27] (Figure 2). The tail has a higher concentration of proteins involved in motility. The sperm membranes of both bulls and bucks consist of a lipid bilayer rich in phospholipids and cholesterol [28]. A key structural feature of this organization is the presence of cholesterol- and sphingolipid-enriched lipid rafts, which serve as dynamic platforms for protein localization and signaling during capacitation and fertilization. Studies in mice and humans have shown that, following the acrosome reaction, proteins such as IZUMO1 and SPACA6 cluster within these raft microdomains, facilitating their interaction with oocyte receptors, including JUNO and CD9. Although direct evidence in farm animal species remains limited, these mechanisms are likely conserved and warrant further experimental verification in bulls and bucks to confirm their functional relevance in mammalian fertilization [29]. Although the exact protein mass per sperm cell remains unclear, functional studies in mouse models show that IZUMO1 levels below 16% of the normal level result in failed fertilization, emphasizing its critical role [30]. Understanding the spatial distribution of these proteins is essential for clarifying how gametes recognize and interact with each other. Equally important is the raft-mediated organization of these proteins, which plays a critical role in the precise control of gamete recognition and fusion mechanisms.

Cholesterol molecules play essential roles in sperm membranes by modulating both membrane fluidity and structural stability [31,32]. Within the lipid bilayer, cholesterol intercalates between phospholipid molecules, reducing excessive packing of fatty acid chains and thereby maintaining an optimal level of membrane order [33]. The rigid steroid ring of cholesterol restricts the mobility of phospholipid tails, which broadens and smooths the phase transition rather than inducing abrupt changes between liquid-crystalline and gel states [34,35]. Through this dual effect, cholesterol helps the membrane remain flexible at low temperatures while preserving integrity and preventing leakage or phase separation. Cholesterol is the principal sterol in sperm membranes across mammalian species, playing a critical role in maintaining their functional stability during fertilization and cryopreservation.

Glycoproteins and glycolipids with carbohydrate chains are present in the sperm membrane surface. These molecules are crucial for cell recognition and interactions during fertilization. Specifically, carbohydrate chains on glycoproteins and glycolipids on the sperm membrane act as ligands that bind to lectins (carbohydrate-binding proteins), which are crucial for sperm–egg recognition, binding, and subsequent fusion of the sperm and egg [36]. The acrosome, located at the tip of the sperm head, contains enzymes such as hyaluronidase and acrosin that enable the sperm to penetrate the egg’s protective layer during fertilization. While the acrosome is not directly responsible for flagellar movement, proteins like dynein and kinesin regulate movement and energy. The ATP produced by the mitochondria in the sperm midpiece is vital for flagellar movement and energy production, ensuring the swimming efficiency of sperm toward the egg and allowing the sperm to undergo the necessary reactions for fertilization [37,38].

Bulls and bucks display differences in sperm morphology, potentially influencing their ability to navigate the female reproductive tract. Variations in the presence of surface proteins and receptors on the sperm membrane can significantly affect the sperm’s ability to recognize and bind to the egg [39]. Species-specific differences may exist in the molecular mechanisms that assist the interaction between sperm and eggs during fertilization. Sperm motility and viability can be affected by any changes in membrane properties. In addition, environmental factors and diet can contribute to variations in the membrane composition, potentially affecting fertility [40]. For instance, dietary lipids, including omega-3 polyunsaturated fatty acids (PUFAs), are vital in the phospholipid bilayer of sperm membranes, enhancing membrane fluidity and resistance to oxidative stress. For this reason, nutrition strategies targeting membrane lipid composition represent a promising approach to optimizing male reproductive performance [41]. However, some studies have reported inconsistent effects of PUFA supplementation, suggesting that excessive or unbalanced intake may increase the susceptibility of sperm membranes to lipid peroxidation, thereby compromising sperm function and freezability. These inconsistencies may be attributed to differences in animal species, lipid sources, or the oxidative environment, indicating that the relationship between dietary lipids, membrane composition, and sperm functionality is complex and context-dependent.

### 2.2. Membrane Proteins

Furthermore, membrane-associated proteins play an equally critical role in fertilization. The ZP3 receptor, integrins, hyaluronidase, IZUMO, lectins, and gangliosides on the sperm membrane that mediate the initial interaction with the egg are critical for successful fertilization [42]. Variations in these proteins may lead to differences in fertilization efficiency between species, particularly the ZP3 receptor, which binds to ZP3, initiating the acrosome reaction; integrins bind to ZP1/ZP2 proteins, aiding in sperm adhesion to the zona pellucida; hyaluronidase degrades hyaluronic acid in the cumulus, enabling sperm to reach the zona pellucida; and lectins facilitate sperm–egg recognition and binding via carbohydrate interactions [43,44]. A better understanding of the molecular anatomy of the sperm membranes and their related components is instrumental for uncovering information about variables that influence sperm physiology and fertility [45]. Although bulls and bucks share fundamental similarities, species-specific differences in sperm membrane composition and function may contribute to variations in reproductive success, which is depicted in Table 1 [46].

## 3. Sperm Cell Membrane and Freezability of Mammalian Sperm

### 3.1. Cryoprotectant Mechanisms

The cell membrane of mammalian sperm plays a crucial role in sperm freezability and the success of cryopreservation depends on maintaining the integrity and functionality of this membrane [10]. Freezing and thawing processes during and after sperm cryopreservation challenge membrane structures, with variations between sperm membranes of different mammalian species impacting their responses to cryopreservation. The structural integrity, fluidity, and functionality of sperm membranes depend on the sufficient presence of lipids and proteins, which are essential for reproductive success [47]. There is significant variation in sperm membrane composition and properties among mammalian species, requiring specific extenders for optimal sperm freezability [48,49].

Typical extenders contain buffers, energy sources, cryoprotectants, proteins/lipids, and antibiotics. Extenders must safeguard membrane integrity, sustain fluidity, improve cryoprotectant permeability, and ensure sperm retains its viability and functionality after thawing [50]. Sperm membranes are particularly susceptible to oxidative stress during cryopreservation, as reactive oxygen species (ROS) can initiate lipid peroxidation in PUFAs, disrupting membrane structure, impairing motility, and reducing fertilization capacity. Understanding species-specific differences in sperm membrane composition is essential for designing effective cryopreservation protocols. From a practical perspective, extender formulation and cryoprotectant selection should be tailored to each species: for bulls, egg yolk- or milk-based extenders combined with glycerol provide effective protection against freezing-induced membrane damage, whereas bucks benefit more from plant-derived phospholipid or liposome-based extenders with lower glycerol concentrations or alternative permeating cryoprotectants such as ethylene glycol. In addition, controlled cooling rates, gradual equilibration, and optimized thawing procedures help minimize osmotic and thermal stress, reduce ROS-induced damage, and improve post-thaw sperm functionality, motility, and fertilization potential. Integrating these strategies ensures that cryopreservation protocols are both effective and species-specific, supporting ART.

The lipid composition and fluidity of the sperm membrane are critical factors influencing sperm quality, including motility, acrosome reaction, overall fertilization potential, and freezability [49]. Sperm membranes rich in fatty acids, which make up the membrane’s phospholipids due to higher concentrations of PUFAs and monounsaturated fatty acids (MUFAs), may tend to be more flexible and are better able to withstand the stresses of freezing and thawing [44]. Also, sperm membranes with high levels of saturated fatty acids, such as palmitic and stearic acids, may have a more rigid structure, which can make the sperm more susceptible to damage during freezing [51]. Additionally, cholesterol plays a key role in maintaining membrane stability. As a critical component of the lipid bilayer, cholesterol interacts with phospholipids to regulate membrane fluidity and rigidity.

### 3.2. Freeze–Thaw Damage and ROS Effect

**Figure 3:**
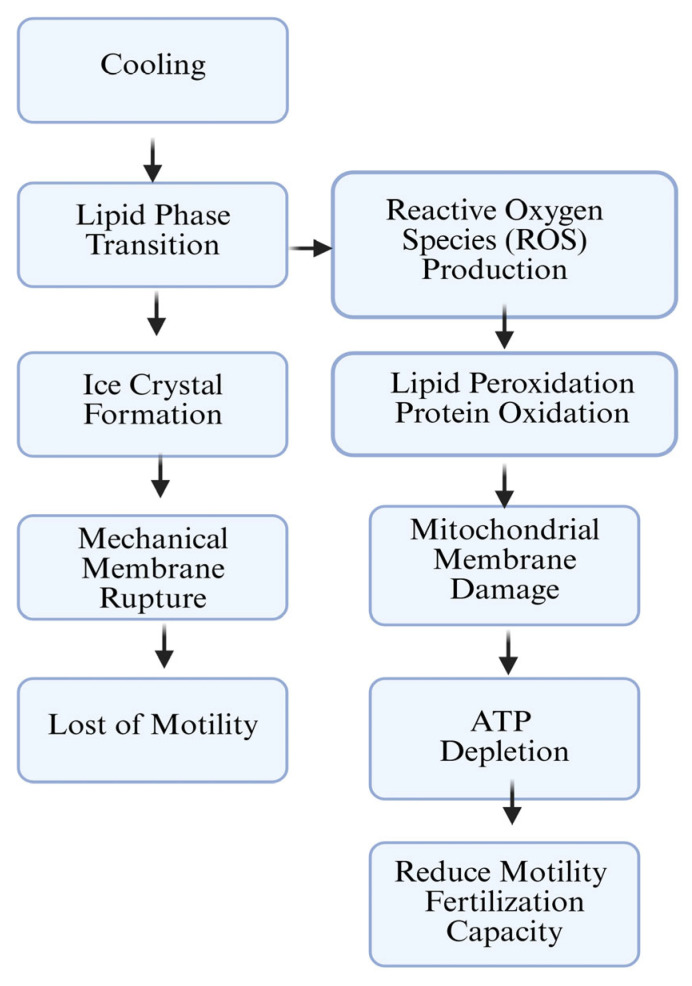
Mechanisms of freeze–thaw damage; schematic representation of the main cellular and molecular events associated with cryoinjury during the freeze–thaw process. Cooling induces lipid phase transition and ice crystal formation, leading to mechanical membrane rupture and loss of motility. Simultaneously, reactive oxygen species (ROS) are produced, causing lipid peroxidation, protein oxidation, and mitochondrial membrane damage. These processes result in ATP depletion, reduced motility, and decreased fertilization capacity.

Although sperm cryopreservation is widely practiced, the specific role of sperm membrane structure and function in determining fertility and freezability remains poorly understood. Variability in lipid composition, phase transition temperature (Tm), and the cholesterol–phospholipid ratio represents key factors that influence membrane stability and post-thaw function. Cholesterol helps to balance the membrane fluidity necessary for sperm motility and the structural integrity required for proper function, making it essential for successful fertilization [51,52]. However, high cholesterol levels reduce membrane fluidity, making the membrane too rigid and unable to withstand the formation of ice crystals during the freezing process [51,53]. During freezing, sperm membranes undergo physical and structural changes, particularly as they pass through their phase transition temperature (Tm)—the point at which membrane lipids shift from a fluid, liquid-crystalline state to a more rigid, gel-like phase [54]. This transition reduces membrane flexibility and can increase the risk of cold shock damage, especially in membranes with high cholesterol or saturated fatty acid content [55]. Therefore, understanding and accounting for the specific Tm of sperm membrane lipids is crucial in designing extenders and cryopreservation protocols that minimize thermal stress and preserve membrane integrity (Figure 3).

Extenders may contain cryoprotectants to counteract the potential effects of cholesterol [56]. Glycolipids are components of the plasma membrane, making them particularly susceptible to cold shock injury [57]. Further characterization of the glycolipid content and composition of sperm plasma membranes from species with varying cold shock sensitivities, particularly through the comparative profiling of specific gangliosides such as GM1, GM3, and GD1a, may provide valuable insights into their roles in modulating membrane phase behavior and enhancing resistance to cold-induced damage. [58]. There are differences in the composition and thermotropic phase glycolipids (the behavior of glycolipids in response to changes in temperature) of sperm among different animal species, which may be related to the greater tolerance of cooling [31,52].

Cryobiology is a multidisciplinary field that studies the physical and biological characteristics of organisms, including cells and tissues [58]. It is critical to livestock production as it enables and accelerates the spread of genetic diversity and facilitates the worldwide distribution of genetically superior animals through the use of cryopreserved sperm in ART. For this reason, cryoprotectants can be used to protect sperm cells during freezing; cryoprotectants can be divided into two main categories, namely permeating and non-permeating cryoprotectants [59,60]. Both types of cryoprotectants have different roles during freezing and thawing [61]. Permeating cryoprotectants such as glycerol, DMSO, ethylene glycol, propanediol, and trehalose penetrate the sperm cell membrane and interact with intracellular components [62]. These molecules mitigate ice crystal formation and reduce osmotic and mechanical stress during freezing. In contrast, non-permeating cryoprotectants like egg yolk, milk proteins, polyvinyl alcohol, and sodium chloride remain outside the cell and provide protection externally [63]. These substances help prevent the formation of ice crystals and preserve cell viability [64]. The selection of cryoprotectants, including glycerol, egg yolk, and DMSO, and their concentrations may vary based on the species, including between bulls and bucks [65]. Thus, differences in sperm membranes, including lipid composition, protein structure, acrosomal integrity, and antioxidant defense, can significantly influence sperm cryobiology [55,66].

Enhancing cryopreservation methods through innovating appropriate cryoprotectants, adjusting the cooling rate, thawing protocols, and addressing species-specific needs, as well as comprehending the distinct requirements of each species, necessitates a comprehensive understanding of cryobiological principles and species-specific sperm physiology [67]. Successful cryopreservation depends on the type and concentration of cryoprotectants, which must balance osmotic tolerance with toxicity. It also requires precise control of cooling rates to avoid phase transitions and intracellular ice formation, as well as optimized thawing rates to minimize recrystallization damage. In particular, the membrane fluidity and cryo-tolerance of the sperm are included in several factors, including membrane lipid compositions, cholesterol–phospholipid ratios, and surface protein profiles. Therefore, adjusting cryopreservation protocols to account for these interspecies variations is crucial for maintaining acrosomal integrity, mitochondrial activity and, ultimately, post-thaw fertility and motility.

## 4. Conclusions

This minireview highlights the importance of sperm membrane structure and function in relation to male fertility and sperm cryopreservation efficiency across mammalian species. It emphasizes species-specific anatomical and physiological features, particularly in bulls and bucks, that influence reproductive performance. The review also explores key molecular and biophysical traits of sperm membranes, such as fluidity, lipid composition, and phase behavior, that contribute to fertility outcomes. Furthermore, the impact of intrinsic (e.g., genetics) and extrinsic (e.g., diet, environment) factors on sperm membrane dynamics is addressed. Collectively, this knowledge provides a framework for improving reproductive technologies in livestock through innovative and transdisciplinary research.

Future scientific advancements are expected to uncover molecular signatures within sperm membranes that serve as reliable biomarkers of fertility and freezability. An improved understanding of lipid–protein interactions and membrane remodeling during capacitation may lead to more precise fertility diagnostics. Technological innovations—for instance, membrane-targeted cryoprotectants and lipid supplementation strategies—hold promise for enhancing sperm preservation protocols. Comparative studies across species will likely refine our ability to tailor cryopreservation methods based on membrane characteristics. Ultimately, these developments could support more efficient genetic dissemination and sustainable livestock production systems, particularly in economically significant farm animals like cattle and goats, to ensure sustainable global food security.

## Figures and Tables

**Table 1:** Comparative compositions of lipids and proteins in the sperm membranes of bulls and bucks.

Component	Bull Sperm Membrane	Buck Sperm Membrane
Fatty Acids	Predominantly saturated and monounsaturated fatty acids; typically, 60–70% of total lipids. PUFAs represent ~20–25%.	Enriched in PUFAs such as arachidonic acid and docosahexaenoic acid; overall unsaturation index higher than in bulls, contributing to greater membrane fluidity.
Proteins	Major proteins include adhesion molecules (integrins, CD9), enzymes (hyaluronidase), and ZP3 receptor proteins.	Similar protein repertoire but relatively higher levels of sperm adhesins, suggesting species-specific modulation of sperm–egg interaction.
Cholesterol	Cholesterol–phospholipid ratio ≈ 0.45–0.50; contributes to membrane rigidity and stability.	Cholesterol–phospholipid ratio ≈ 0.30–0.35 (lower than bull), promoting a more fluid membrane and facilitating the acrosome reaction.
Glycolipids	Dominated by GM3 gangliosides, associated with membrane stabilization and sperm–egg recognition.	Enriched in GD3 gangliosides, potentially enhancing signal transduction during fertilization.
Glycoproteins	Contains ZP3-binding glycoproteins and sperm adhesins, key for zona pellucida binding.	Similar glycoproteins but higher sperm adhesin abundance, possibly improving sperm–egg interaction efficiency.
Membrane Phase Transition (Tm)	Estimated Tm ≈ 22–25 °C (reflecting higher saturation and cholesterol).	Estimated Tm ≈ 15–18 °C, indicating greater unsaturation and fluidity; quantitative confirmation needed.

## Data Availability

No data was used for the research described in the article.
